# A Composite Indicator of Inflammation and Nutritional Status for Predicting Mortality After CABG: The CRP-to-Albumin Ratio

**DOI:** 10.3390/nu18111721

**Published:** 2026-05-28

**Authors:** Samet Yımaz, Nihat Söylemez, Mustafa Ekici, Mahmut Yılmaz, Ali Orçun Sürmeli, Burak Toprak, Özkan Karaca, Rıdvan Bora, Mehmet Ballı, Serdar Keçeoğlu

**Affiliations:** 1 Department of Cardiology, Başkent University Adana Research Center, Adana 01250, Turkey; sametyilmaz.dr@gmail.com; 2 Department of Cardiology, Mersin City Education and Research Hospital, Mersin 33240, Turkey; drnihatsylmz@gmail.com (N.S.); myilmaz510@hotmail.com (M.Y.); md.ozkrc@gmail.com (Ö.K.); dr.ridvanbora@outlook.com (R.B.); dr_mehmetballi@hotmail.com (M.B.); serdarkeceoglu@gmail.com (S.K.); 3 Department of Emergency Medicine, Mersin Provincial Health Directorate, Mersin 33110, Turkey; dr.mustafaekici@hotmail.com; 4 Department of Cardiovascular Surgery, Mersin City Education and Research Hospital, Mersin 33240, Turkey

**Keywords:** C-reactive protein, albumin, C-reactive protein-to-albumin ratio, coronary artery bypass grafting, mortality

## Abstract

**Background:** The C-reactive protein-to-albumin ratio (CAR) has emerged as a composite biomarker reflecting both systemic inflammation and nutritional status. This study investigated the prognostic value of preoperative and postoperative CAR in predicting early mortality following coronary artery bypass grafting (CABG). **Methods:** This retrospective study included 273 patients who underwent isolated CABG. Patients were categorized as mortality (+) (Ex group, *n* = 41) and mortality (−) (Non-Ex group, *n* = 232). Preoperative and postoperative CAR values were calculated and compared between groups. Independent predictors of mortality were evaluated using logistic regression analysis. Discriminative performance was assessed using receiver operating characteristic (ROC) curve analysis. **Results:** Early mortality occurred in 41 patients (15.0%). Both preoperative and postoperative CAR values were significantly higher in the mortality (+) group compared with the mortality (−) group. In multivariable logistic regression analysis, postoperative creatinine remained independently associated with early mortality, whereas neither preoperative nor postoperative CAR retained independent statistical significance after adjustment for clinical variables and renal function despite moderate discriminative performance in crude and ROC-based analyses. ROC analysis showed that CAR demonstrated moderate discriminative ability for mortality prediction, with postoperative CAR showing better performance than preoperative CAR. **Conclusions:** Although CAR demonstrated moderate discriminative performance for early mortality after CABG in unadjusted analyses, it did not independently predict mortality after adjustment for clinical variables and renal function. Therefore, CAR may be more appropriately interpreted as a supportive inflammation-based risk stratification marker rather than a stand-alone prognostic predictor.

## 1. Introduction

Coronary artery disease continues to be one of the leading causes of cardiovascular mortality and morbidity both worldwide and in our country [1]. In advanced diffuse coronary artery disease, surgical revascularization is accepted as a strong treatment option, particularly due to its long-term survival advantage in multivessel disease, left main coronary artery involvement, and complex anatomical lesions [2]. Nevertheless, early postoperative complications and mortality after coronary artery bypass grafting (CABG) surgery still remain among the main challenges in clinical practice, and risk prediction is of critical importance for optimizing perioperative management [1].

The determinants of mortality after CABG are multidimensional and cannot be explained solely by coronary anatomy or surgical technique [3]. Patient-related characteristics such as age, concomitant comorbidities, left ventricular function, renal reserve, metabolic status, and the capacity to mount a systemic inflammatory response, in combination with the surgical stress response, cardiopulmonary bypass (CPB)-related inflammation, and organ perfusion disturbances, markedly shape the risk of mortality [1]. Therefore, although risk scoring systems such as EuroSCORE II and STS have become widely used in clinical practice, it has been shown that these scores do not perform with the same accuracy across all populations, and that individual-level risk discrimination may be insufficient, particularly in heterogeneous patient groups [4]. Moreover, current risk prediction models are largely based on demographic, clinical, and procedural variables, and do not directly incorporate dynamic biological processes such as systemic inflammation and metabolic reserve. As a result, there is increasing interest in investigating whether easily accessible biomarkers may provide complementary biological information alongside existing clinical risk stratification frameworks. In this context, composite indices such as CAR may reflect biological dimensions of perioperative stress and inflammation that are not directly represented within traditional clinical scoring systems.

The pathophysiological basis of CABG surgery includes CPB-related “sterile inflammation,” endothelial activation, complement cascade triggering, cytokine release, and increased oxidative stress [5]. This process also predisposes to impaired tissue perfusion, microcirculatory dysfunction, and multi-organ involvement [5]. The relationship between the degree of inflammatory activity and the risk of postoperative complications becomes more pronounced, particularly in major clinical outcomes such as the intensive care course, development of infection, renal dysfunction, hemodynamic instability, and mortality [6].

One of the biomarkers reflecting the clinical manifestations of inflammation is C-reactive protein (CRP), which has long been used as a quantitative indicator of the systemic inflammatory response after cardiovascular surgery [7]. However, while CRP alone reflects the inflammatory burden, it does not directly represent other critical biological domains determining mortality, such as physiological reserve and nutritional status [7]. In this context, serum albumin level is an indirect indicator of both nutritional status and inflammation through the negative acute-phase response, and many studies have emphasized that hypoalbuminemia may be associated with poor clinical outcomes after surgery [8].

In recent years, the “composite index” approach has gained importance to overcome the limitations of single biomarkers. The CRP-to-albumin ratio (CAR) has emerged as a practical parameter that combines inflammatory activity and nutritional/reserve status within the same mathematical framework [9]. The prognostic potential of CAR has been reported particularly in intensive care patients, sepsis, malignancies, and various cardiovascular clinical scenarios [10]. In the field of cardiac surgery, inflammation-based biomarkers such as CRP, NLR, PLR, and composite indices have been increasingly investigated for their prognostic value in postoperative outcomes, including mortality and major complications [11]. Several studies have also explored the role of CAR in cardiovascular and surgical populations, including patients undergoing off-pump CABG and transcatheter interventions, suggesting a potential association with adverse outcomes [12,13]. However, these findings are heterogeneous and often limited by differences in study design, patient selection, timing of biomarker assessment, and outcome definitions. Therefore, the exact clinical position of CAR within the context of CABG-specific risk stratification remains incompletely defined. Although inflammation-based biomarkers have increasingly attracted attention in CABG populations, the precise clinical role of CAR within contemporary perioperative risk assessment remains incompletely defined. In particular, it remains uncertain whether perioperative CAR measurements provide clinically meaningful information beyond routine clinical assessment and established perioperative risk factors [7,8].

In this context, for earlier and more accurate risk stratification in terms of post-CABG mortality, personalization of intensive care strategies, and a proactive approach to complications, there is a need for a simple, rapid, and cost-effective marker. The CRP-to-albumin ratio is one of the “composite biomarker” candidates that may meet this need. However, for CAR to be a clinically applicable indicator, its association with post-CABG mortality should be demonstrated not only through crude comparisons but also through multivariable analyses, discriminative performance, and appropriate cut-off values.

The aim of this study was to evaluate the prognostic role of preoperative and postoperative CRP-to-albumin ratio in patients undergoing CABG, and to determine whether CAR demonstrates clinically meaningful discrimination of mortality risk when evaluated together with conventional clinical risk factors, with particular emphasis on the comparative value of perioperative measurements.

## 2. Materials and Methods

### 2.1. Data Collection

#### 2.1.1. Study Design

This study was designed as a single-center, retrospective, observational cohort study. In addition, the study was conducted and reported in accordance with the REporting of studies Conducted using Observational Routinely-collected health Data (RECORD) statement, which extends the STROBE guidelines for studies using routinely collected health data. The study evaluated the predictive performance of the preoperative and postoperative C-reactive protein/albumin ratio for early mortality in patients undergoing coronary artery bypass grafting (CABG). Due to the retrospective design, no additional interventions were applied to the patients, and all data were analyzed based on records obtained during routine clinical follow-up. The primary endpoint of the study was early mortality, and mortality status was classified as Ex (patients experienced mortality) and Non-Ex (patients did not experience mortality). Early mortality was defined as in-hospital mortality occurring during the index hospitalization period following CABG surgery. Postoperative mortality was defined as in-hospital mortality occurring during the index hospitalization period following CABG surgery. Thirty-day mortality after discharge was not evaluated in the present study. Reporting of the study was planned in accordance with the international reporting guidelines recommended for observational studies (STROBE).

#### 2.1.2. Data Collection

The study population was derived from patients who underwent isolated CABG surgery within the predefined study period. The study period covered patients operated between January 2021 and August 2025. Because early postoperative mortality represented a relatively infrequent outcome, an event-enriched analytic sampling strategy was applied to improve statistical efficiency for mortality-related analyses. Accordingly, mortality cases were included as comprehensively as possible, whereas non-mortality cases were selected at a predefined ratio to construct the final analytic cohort. Formal propensity-score matching was not performed, and the sampling process was based primarily on event enrichment to improve statistical efficiency for mortality-related analyses. Inclusion criteria were age ≥ 18 years, having undergone isolated CABG surgery, definite documentation of mortality status, and availability of both CRP and albumin measurements in the preoperative and postoperative periods to enable CAR calculation. Exclusion criteria were combined cardiac surgery (e.g., valve surgery or aortic surgery concomitant with CABG), active infection or sepsis, known malignancy, chronic inflammatory/autoimmune disease, advanced-stage liver failure (clinical conditions that may markedly affect albumin synthesis), hematological malignancy/active hematological disease, use of immunosuppressive therapy, missing or unverifiable records, and patients lacking either CRP or albumin measurements. The aim of this approach was to reduce the confounding effect of conditions that may primarily influence CRP and albumin levels due to infection, malignancy, or severe systemic diseases, and to more accurately evaluate the prognostic value of CAR.

Data were obtained from the hospital’s electronic medical record system, operative reports, and intensive care follow-up forms. Demographic characteristics (age, sex), major comorbidities (Diabetes mellitus [DM], Hypertension [HT]), perioperative variables (cardiopulmonary bypass time, cross-clamp time), and laboratory parameters (preoperative and postoperative CRP and serum albumin levels) were recorded. CAR was calculated for each patient separately for the preoperative and postoperative periods by dividing CRP by the albumin level. CAR was calculated separately for the preoperative and postoperative periods by dividing serum CRP values (mg/L) by serum albumin values (g/L), resulting in a dimensionless ratio. The CRP/albumin ratio was considered a composite indicator integrating the level of inflammatory response and the nutritional/reserve status within a single parameter. During data collection, the timing of measurements was defined according to the period in which laboratory tests were performed based on the routine clinical protocol; preoperative measurements were accepted as the last values obtained within routine preoperative evaluation, and postoperative measurements were accepted as values obtained during early postoperative clinical follow-up. Specifically, postoperative CRP and albumin measurements used for CAR calculation were obtained at the 24th hour after surgery as part of routine postoperative laboratory assessment.

To increase the reliability of the study data, a data cleaning and quality control process was applied. First, duplicate records were removed, clinically meaningless or biologically implausible values were verified by returning to the data source, and records that could not be verified were excluded. Negative or extreme values in time variables were screened, and possible input errors in laboratory measurements were checked. For patients with missing data, records were re-screened; cases in which missingness could not be resolved and that did not include key variables (particularly CRP and albumin measurements) were excluded. Thus, the final sample used for analysis consisted of patients who fully met the inclusion and exclusion criteria.

### 2.2. Data Analysis

#### 2.2.1. Statistical Analysis

The statistical analysis plan was structured in advance to evaluate an outcome of high clinical importance such as mortality after CABG. A two-sided hypothesis testing approach was adopted in the analyses, and the level of statistical significance was set at *p* < 0.05. Distributional characteristics of continuous variables were visually assessed using histograms and Q–Q plots, and normality was confirmed using the Shapiro–Wilk test. Continuous variables with normal distribution were reported as mean ± standard deviation, whereas non-normally distributed variables were reported as median (interquartile range; IQR). Categorical variables were presented as numbers and percentages.

To increase the analytical power given the number of mortality events (Ex), an event-enriched approach was adopted during sample construction. Accordingly, the mortality proportion observed in the analytic cohort does not represent the natural mortality prevalence of an unselected CABG population. Rather, this strategy was intentionally used to improve statistical efficiency for mortality-related analyses and to enable more stable discrimination modeling. Nevertheless, this enrichment approach may limit direct generalizability of prevalence-dependent performance measures to routine clinical populations. In this context, patients who experienced mortality were included as much as possible, and patients who did not experience mortality (Non-Ex) were selected using an approximately 1:5 event-enriched sampling ratio to construct the final analytic cohort. This method is preferred in rare outcomes (particularly mortality) to increase statistical power and to enable more stable multivariable modeling. However, considering that this design may increase the Ex proportion compared to the natural population, the results were interpreted not to reflect mortality prevalence but to evaluate the association between CAR and mortality and its discriminative performance. Therefore, it was acknowledged that caution is required when generalizing prevalence-sensitive measures such as PPV and NPV to the general population; in contrast, prevalence-independent performance measures such as AUC were considered the primary evaluation criteria.

For comparisons of continuous variables between the Ex and Non-Ex groups, the independent samples Student *t*-test was used for parameters meeting the normality assumption. For parameters not meeting the normality assumption, the Mann–Whitney U test was preferred. For between-group comparisons of categorical variables, the chi-square test was used; Fisher’s exact test was applied in cases where the expected cell count was below 5. The purpose of these comparisons was to preliminarily evaluate clinical and laboratory variables that might be associated with mortality and to identify potential confounders for further analyses.

The performance of the CRP/albumin ratio in predicting mortality was analyzed separately for both the preoperative and postoperative periods. The association between CAR and mortality was first evaluated using a continuous variable approach; subsequently, to ensure interpretability for clinical use, cut-off values were determined using Receiver Operating Characteristic (ROC) curve analysis. Discriminative performance in ROC analysis was expressed as AUC (Area Under the Curve), and 95% confidence intervals were calculated. The optimal cut-off value was selected based on the Youden index (J = sensitivity + specificity − 1). According to the identified threshold value, CAR was classified as low and high; based on this classification, sensitivity, specificity, accuracy, positive predictive value (PPV), and negative predictive value (NPV) were calculated. To increase the reliability of ROC analysis outputs, internal validation of AUC was performed using bootstrap-based sampling, and the stability of performance measures was tested.

Logistic regression models were constructed to evaluate the independent association between CAR and mortality. First, variables potentially associated with mortality were assessed using univariable logistic regression analysis; subsequently, multivariable logistic regression analysis was performed by including covariates considered clinically meaningful and variables found to be associated in univariable analysis. During the modeling process, the risk of multicollinearity was assessed using the Variance Inflation Factor (VIF). To reduce the risk of model overfitting given the limited number of mortality events, covariate inclusion was restricted to clinically relevant variables and parameters demonstrating potential association in univariable analyses. In addition, variables with substantial biological overlap or potential collinearity were not simultaneously incorporated into the same multivariable model. Accordingly, because CAR mathematically incorporates both CRP and albumin, these individual parameters were not simultaneously entered into the adjusted models together with CAR in order to avoid unstable coefficient estimation and multicollinearity-related distortion. In addition, considering that the number of mortality events may be limited, the risk of overfitting was reduced, and the events-per-variable principle was considered in model construction. Regression results were reported as Odds Ratio (OR) and adjusted Odds Ratio (aOR) with 95% confidence intervals. Model fit was evaluated using goodness-of-fit tests and residual analyses, and classification performance was re-evaluated based on ROC-AUC. Thus, the aim was to demonstrate the prognostic value of CAR not only based on crude group comparisons but also on the basis of independent predictability through a multivariable approach.

#### 2.2.2. Software

All statistical analyses were performed using Python (version 3.12). The pandas and numpy libraries were used for data processing, organization, and cleaning. Descriptive statistics and hypothesis tests were conducted using scipy, whereas model-based analyses such as logistic regression were performed using statsmodels. ROC analysis, AUC calculations, cut-off determination, and evaluation of classification performance were performed using the scikit-learn package. A code-based approach was preferred throughout the analysis workflow, and reproducibility principles were ensured by standardizing the applied data cleaning steps and the analysis pipeline.

## 3. Results

In the Section 3, baseline clinical characteristics, perioperative variables, and laboratory parameters were compared between the Ex and Non-Ex groups. Additionally, the prognostic performance of preoperative and postoperative CAR for predicting early mortality was evaluated through regression modeling and ROC curve analyses.

The baseline comparison presented in Table 1 revealed that EF was significantly lower in the Ex group than in the Non-Ex group (50.0 [46.0–55.0] vs. 55.0 [50.0–57.0], *p* = 0.029). In addition, LDL levels were significantly higher in the Ex group compared to the Non-Ex group (116.6 [83.9–145.0] vs. 103.3 [73.0–129.3] mg/dL, *p* = 0.044). In addition, cardiopulmonary bypass and aortic cross-clamp durations were significantly longer in the Ex group than in the Non-Ex group (both *p* < 0.001). (Table 1).

To improve interpretability, preoperative and postoperative laboratory analyses were evaluated separately. The preoperative laboratory findings in Table 2 demonstrated that preoperative CRP levels were significantly higher in the Ex group compared to the Non-Ex group (62.0 [21.0–123.8] vs. 31.7 [13.0–68.0] mg/L, *p* = 0.007). Preoperative albumin was significantly lower in the Ex group (32.0 [29.0–35.0] vs. 34.0 [31.3–37.0] g/L, *p* = 0.009), while preoperative CAR was significantly higher in the Ex group (1.85 [0.67–4.33] vs. 0.91 [0.39–2.19], *p* = 0.003). In addition, preoperative urea (45.0 [33.5–70.0] vs. 35.0 [27.0–46.8] mg/dL, *p* = 0.001) and preoperative creatinine (1.07 [0.82–1.57] vs. 0.88 [0.76–1.04] mg/dL, *p* = 0.005) were significantly higher in the Ex group, whereas preoperative GFR was significantly lower (66.4 [42.0–91.0] vs. 84.0 [66.0–101.0] mL/min/1.73 m^2^, *p* = 0.009). Moreover, preoperative hemoglobin (12.6 [10.9–13.7] vs. 13.3 [12.3–14.3] g/dL, *p* = 0.016) and preoperative hematocrit (38.7 [33.4–41.1] vs. 40.4 [37.1–43.4] %, *p* = 0.023) were significantly lower in the Ex group. The Ex group also exhibited significantly higher preoperative WBC counts (10.1 [7.8–12.6] vs. 8.7 [7.3–10.6] × 10^9^/L, *p* = 0.039) and significantly higher preoperative RDW values (14.9 [14.2–16.2] vs. 14.3 [13.6–15.2] %, *p* = 0.006) (Table 2).

The postoperative laboratory parameters in Table 3 indicated that postoperative CRP was significantly higher in the Ex group than in the Non-Ex group (94.0 [40.0–153.0] vs. 42.5 [21.0–83.1] mg/L, *p* < 0.001). Postoperative albumin levels were significantly lower in the Ex group (27.0 [24.8–30.0] vs. 29.0 [27.0–32.0] g/L, *p* = 0.001), while postoperative CAR was significantly higher (3.39 [1.55–6.18] vs. 1.44 [0.73–2.93], *p* < 0.001). In addition, postoperative urea (59.0 [39.0–97.0] vs. 34.0 [25.0–47.0] mg/dL, *p* < 0.001) and postoperative creatinine (1.63 [1.00–2.62] vs. 0.89 [0.76–1.06] mg/dL, *p* < 0.001) were significantly higher in the Ex group, whereas postoperative GFR was significantly lower (46.0 [24.9–74.0] vs. 82.2 [64.0–99.0] mL/min/1.73 m^2^, *p* < 0.001). Furthermore, postoperative hemoglobin was significantly lower in the Ex group (9.8 [8.5–11.2] vs. 10.6 [9.7–11.8] g/dL, *p* = 0.018). The Ex group also showed significantly higher postoperative WBC counts (13.0 [10.4–16.5] vs. 11.4 [9.3–13.8] × 10^9^/L, *p* = 0.009), significantly lower postoperative platelet counts (158.0 [106.0–204.0] vs. 186.0 [142.0–239.0] × 10^9^/L, *p* = 0.024), and significantly higher postoperative RDW values (15.4 [14.6–16.9] vs. 14.4 [13.7–15.3] %, *p* < 0.001). Moreover, postoperative IG was significantly higher in the Ex group (0.90 [0.60–1.50] vs. 0.60 [0.40–0.98] %, *p* = 0.002), while postoperative lymphocytes were significantly lower (0.90 [0.63–1.24] vs. 1.10 [0.84–1.49] × 10^9^/L, *p* = 0.015) (Table 3).

The univariate logistic regression analysis presented in Table 4 demonstrated that ejection fraction was significantly associated with postoperative mortality (OR 0.949, 95% CI 0.905–0.996, *p* = 0.033). Preoperative creatinine (OR 2.884, 95% CI 1.047–7.941, *p* = 0.040) and postoperative creatinine (OR 2.813, 95% CI 1.466–5.395, *p* = 0.002) were also significantly associated with mortality. In addition, postoperative albumin was inversely associated with mortality risk (OR 0.862, 95% CI 0.791–0.939, *p* < 0.001), whereas postoperative urea was identified as a significant positive predictor of mortality (OR 1.348, 95% CI 1.101–1.652, *p* = 0.004). Moreover, postoperative platelet count was significantly associated with mortality (OR 0.928, 95% CI 0.871–0.988, *p* = 0.020). Although preoperative CAR demonstrated a trend toward association with mortality (OR 2.00, 95% CI 0.91–4.37, *p* = 0.081), neither preoperative nor postoperative CAR reached statistical significance in univariate logistic regression analysis (Table 4).

In the multivariable logistic regression models presented in Table 5, postoperative creatinine remained independently associated with in-hospital mortality (Adjusted OR 3.118, 95% CI 1.560–6.231, *p* = 0.001). Female sex was also significantly associated with mortality in both preoperative and postoperative adjusted models. Although both preoperative and postoperative CAR showed clinically relevant associations in crude and ROC-based analyses, neither preoperative CAR nor postoperative CAR remained statistically significant after adjustment for core clinical variables and renal function (Table 5).

The ROC analysis in Table 6 showed that preoperative CAR had a significant discriminative performance for predicting mortality (AUC 0.676, 95% CI 0.591–0.760, *p* = 0.001), while postoperative CAR demonstrated a higher significant performance (AUC 0.792, 95% CI 0.725–0.859, *p* < 0.001). EF alone also showed significant discrimination (AUC 0.641, 95% CI 0.555–0.726, *p* = 0.006), whereas SYNTAX score alone did not show significant predictive ability (AUC 0.512, 95% CI 0.420–0.604, *p* = 0.812). The combined mini-models also yielded significant results, including Pre CAR + EF (AUC 0.724, 95% CI 0.643–0.805, *p* < 0.001) and Post CAR + EF (AUC 0.823, 95% CI 0.761–0.884, *p* < 0.001). Based on the Youden index, the optimal cut-off value was determined as preoperative CAR ≥ 1.30, yielding sensitivity 63.4% and specificity 67.2%, and postoperative CAR ≥ 2.60, yielding sensitivity 78.0% and specificity 74.6%. In binary risk estimation, Pre CAR ≥ 1.30 was significantly associated with mortality (OR 3.12, 95% CI 1.48–6.56, *p* = 0.003), and Post CAR ≥ 2.60 showed a stronger significant association (OR 6.85, 95% CI 3.20–14.65, *p* < 0.001) (Table 6).

Calibration analysis of the multivariable models is presented in Table 7. The preoperative model demonstrated a calibration slope of 0.91 and an intercept of 0.04, indicating good agreement between predicted and observed outcomes, with a Brier score of 0.136. Similarly, the postoperative model showed a calibration slope of 0.95 and an intercept of 0.02, suggesting adequate calibration and minimal systematic prediction error. The Brier score for the postoperative model was 0.129, reflecting slightly improved overall predictive accuracy compared with the preoperative model. These findings indicate that, in addition to moderate discrimination, both models demonstrated acceptable calibration performance (Table 7).

**Figure 1:**
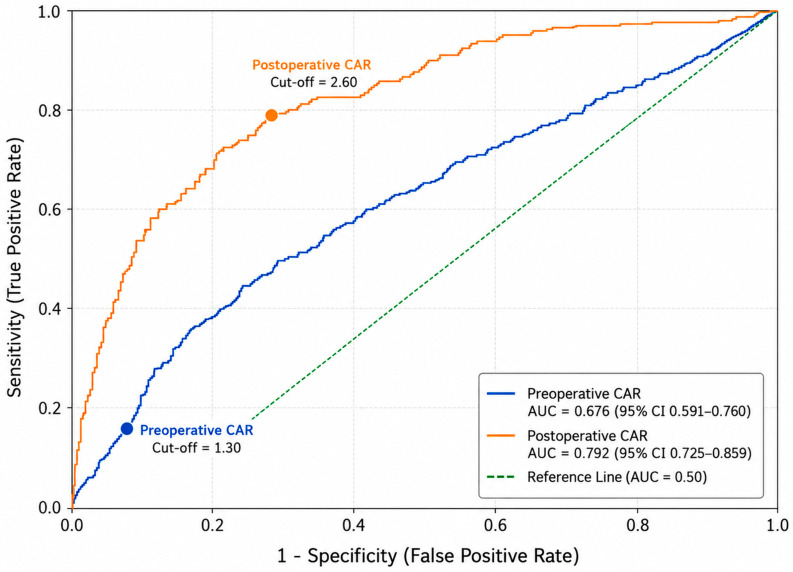
Receiver operating characteristic (ROC) curves of preoperative and postoperative C-reactive protein-to-albumin ratio (CAR) for predicting early postoperative mortality after CABG. The ROC analysis demonstrates the discriminative performance of CAR measured preoperatively and postoperatively in the final analytic cohort (*n* = 273). Postoperative CAR demonstrated superior discriminative ability compared with preoperative CAR. The diagonal dashed line represents the reference line for chance discrimination (AUC = 0.50). The optimal cut-off values derived using the Youden index are highlighted on the curves (preoperative CAR cut-off ≥1.30; postoperative CAR cut-off ≥2.60). AUC values are presented with 95% confidence intervals (preoperative CAR: AUC 0.676 [95% CI 0.591–0.760]; postoperative CAR: AUC 0.792 [95% CI 0.725–0.859]).

Figure 1 presents the ROC curve analysis for preoperative and postoperative CAR in predicting early postoperative mortality after CABG. Postoperative CAR demonstrated superior discriminative performance compared with preoperative CAR, with an AUC of 0.792 (95% CI 0.725–0.859) versus 0.676 (95% CI 0.591–0.760), respectively. The ROC curves were reconstructed using the final analytic dataset, and the optimal cut-off values determined by the Youden index were ≥1.30 for preoperative CAR and ≥2.60 for postoperative CAR. The diagonal dashed line represents the reference line for chance discrimination (AUC = 0.50) (Figure 1).

**Figure 2:**
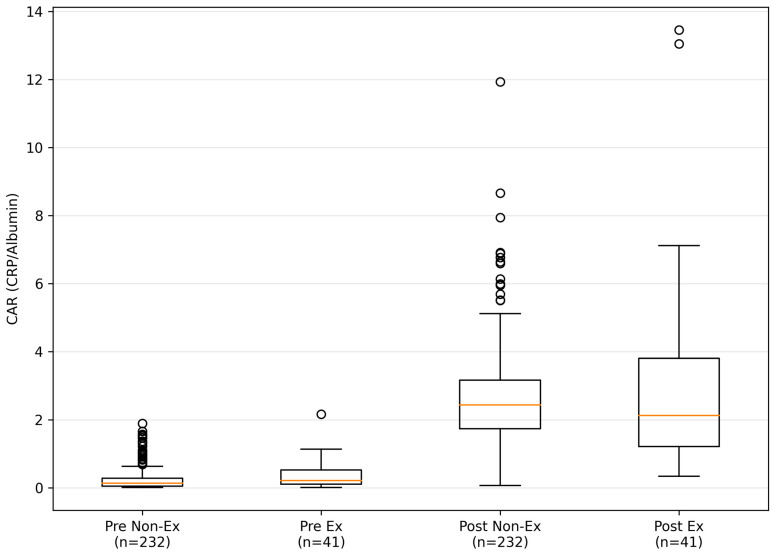
Distribution of preoperative and postoperative C-reactive protein-to-albumin ratio (CAR) according to mortality status. Box-and-whisker plots illustrate CAR values in the Non-Ex and Ex groups before surgery (preoperative) and after surgery (postoperative). The central line represents the median, the box represents the interquartile range (IQR), whiskers indicate the range excluding outliers, and individual circles denote outlier values. Group sample sizes are shown beneath each boxplot (Pre Non-Ex *n* = 232, Pre Ex *n* = 41, Post Non-Ex *n* = 232, Post Ex *n* = 41).

Figure 2 demonstrates the distribution of CAR values across mortality groups in both the preoperative and postoperative periods. Postoperative CAR values were higher than preoperative CAR values in both groups. In addition, CAR values in the Ex group were higher compared to the Non-Ex group, particularly in the postoperative period, where the Ex group showed a broader distribution and higher outlier values (Figure 2).

**Figure 3:**
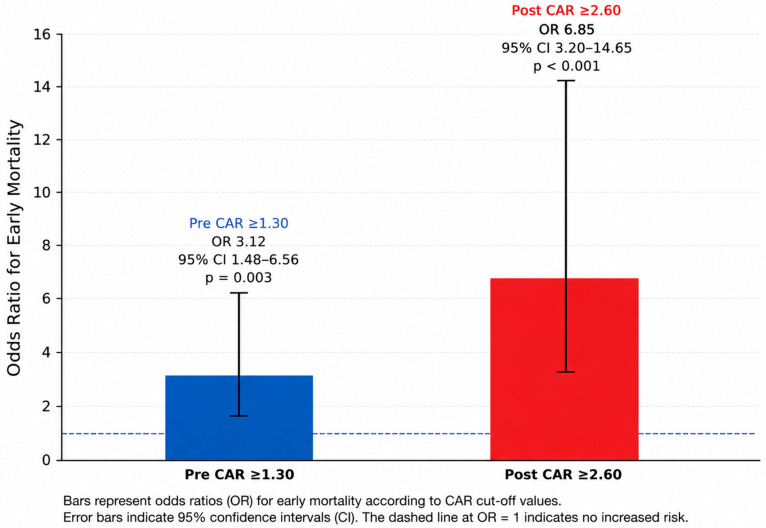
Binary risk estimation according to preoperative and postoperative CAR cut-off values. Bar plots represent odds ratios (ORs) for early mortality according to ROC-derived CAR thresholds (preoperative CAR ≥ 1.30 and postoperative CAR ≥ 2.60). Error bars indicate 95% confidence intervals (95% CI). The dashed horizontal line at OR = 1 represents the reference level for no increased mortality risk.

Figure 3 illustrates the binary risk estimation according to the predefined CAR cut-off values derived from ROC analysis. Preoperative CAR ≥ 1.30 was associated with a significantly increased risk of early mortality (OR 3.12, 95% CI 1.48–6.56, *p* = 0.003), while postoperative CAR ≥ 2.60 demonstrated an even stronger association with mortality risk (OR 6.85, 95% CI 3.20–14.65, *p* < 0.001). Error bars represent 95% confidence intervals for the estimated odds ratios (Figure 3).

## 4. Discussion

In this study, the potential of the preoperative and postoperative CRP/albumin ratio (CAR) to predict early mortality in patients undergoing CABG was evaluated, and CAR, as a composite biomarker integrating inflammatory burden and physiological reserve/nutritional status, demonstrated moderate discriminative ability in crude analyses but did not retain independent prognostic significance after multivariable adjustment. The main findings of our study were that CAR levels were significantly higher in patients who experienced mortality, that CAR—particularly in the postoperative period—was more strongly associated with mortality, and that ROC analysis demonstrated a clinically usable performance for discriminating mortality. However, after adjustment for core clinical variables and renal function, CAR did not remain an independent predictor of mortality, suggesting that its prognostic signal may partly reflect the broader inflammatory, renal, and clinical severity profile of high-risk CABG patients. Therefore, CAR may be more appropriately interpreted as a composite supportive marker reflecting perioperative inflammatory burden, renal dysfunction, hemodilution-related albumin changes, catabolic stress response, and reduced physiological reserve rather than an isolated causal determinant of mortality. Beyond isolated inflammatory activation, CAR may also reflect broader interactions between nutritional status, oxidative stress, metabolic reserve, endothelial dysfunction, and systemic physiological vulnerability. In particular, hypoalbuminemia has increasingly been recognized not only as a marker of malnutrition but also as an indicator of impaired antioxidant defense, frailty, immune dysregulation, and reduced adaptive capacity under acute surgical stress conditions [14]. Recent evidence suggests that inflammation-related nutritional biomarkers may provide integrated information regarding biological reserve, catabolic burden, and systemic resilience, particularly in elderly and critically ill populations [15]. Furthermore, oxidative stress and systemic inflammatory activation are closely interconnected in cardiovascular disease and cardiac surgery, contributing to endothelial injury, microcirculatory dysfunction, organ hypoperfusion, and impaired postoperative recovery [16]. Therefore, CAR should not be interpreted as a disease-specific marker but rather as a multidimensional biological indicator integrating inflammatory, nutritional, metabolic, and physiological stress pathways associated with adverse postoperative outcomes [17]. In particular, the persistence of postoperative creatinine as an independent predictor in multivariable analysis suggests that postoperative renal dysfunction may represent a more dominant determinant of early mortality than inflammation-based composite biomarkers in the perioperative CABG setting. Accordingly, CAR may be more appropriately interpreted as a complementary inflammation-based marker that contributes to perioperative risk stratification rather than a fully independent prognostic determinant. Importantly, the present findings should not be interpreted as suggesting that CAR replaces or outperforms validated perioperative risk models such as EuroSCORE II or STS. These established scoring systems remain the principal frameworks for surgical risk prediction after CABG because they integrate multiple demographic, clinical, hemodynamic, and procedural variables with extensive external validation across large populations. In contrast, the current study was designed primarily to explore the biological and inflammatory correlates of postoperative mortality rather than to develop or validate a novel standalone surgical risk model. Therefore, the potential role of CAR should be interpreted as exploratory and hypothesis-generating, with possible value as a complementary biological marker reflecting perioperative inflammatory and physiological stress rather than as an alternative to established clinical risk stratification systems. In this context, the clinical utility of CAR may derive from its ability to reflect the combined burden of systemic inflammation, nutritional reserve, and perioperative physiological stress within an easily accessible laboratory parameter. These findings suggest that, in the early period after CABG, inflammation and impaired physiological reserve are important components associated with mortality risk, although their relative contribution may vary depending on the overall clinical context.

The pathogenesis of mortality after CABG surgery is not a process that can be explained solely by surgical technique or coronary anatomy; rather, it is a multidimensional clinical outcome that develops through complex biological axes such as systemic inflammatory response, endothelial dysfunction, microcirculatory impairment, and multi-organ involvement [3]. The use of cardiopulmonary bypass can markedly increase postoperative inflammatory burden through blood contact with non-physiological surfaces, complement activation, leukocyte activation, and sterile inflammation that may progress to a cytokine storm [5]. Although an increase in CRP is an expected acute-phase response in this process, CRP level alone does not represent the “risk biology” in all aspects, because one of the determining components of mortality risk is the extent to which the patient’s reserve and recovery capacity can tolerate the inflammatory response [7]. This is precisely where the biological strength of CAR becomes evident: while CAR reflects inflammatory activation through CRP, it simultaneously represents the patient’s nutritional/reserve status and the negative acute-phase response through albumin level within the same index [9]. Therefore, the association between CAR and mortality should be considered not only along the “inflammation” axis, but also together with the effect of the catabolic burden induced by inflammation on the patient’s physiological reserve [12]. While CRP level reflects the severity of inflammation, albumin level represents not only nutritional status but also the inflammation-related negative acute-phase response; thus, CAR may be considered a more comprehensive risk indicator in which two biological processes are combined within a single index [7,8].

The clinical significance of our study lies in the potential contribution of CAR to mortality risk assessment as a simple and accessible biomarker. However, it should be acknowledged that the observed discriminative performance may be partially influenced by the event-enriched design, and therefore, the magnitude of predictive accuracy should be interpreted within this methodological context. CRP and albumin are routinely assessed tests in CABG patients in most centers and do not require additional cost. This makes CAR an applicable tool for early risk stratification, particularly in intensive care practice. From a clinical perspective, postoperative CAR may be interpreted as an easily accessible supportive marker reflecting the magnitude of early postoperative inflammatory and physiological stress. However, because postoperative CAR largely reflects processes that are already partially recognized during routine perioperative monitoring—including inflammation, hemodynamic instability, renal dysfunction, and catabolic response—its clinical utility should not be overstated. Rather than directly guiding therapeutic decisions, elevated postoperative CAR may primarily serve as an adjunctive signal identifying patients who may require closer clinical observation and more careful integration of existing perioperative management strategies [12].

In the literature, the prognostic value of CAR has increasingly been investigated in different clinical scenarios. In particular, CAR has been shown to be associated with mortality in intensive care patients, sepsis, and clinical conditions with a marked inflammatory burden [9,13]. In the field of cardiac surgery, although interest has increased in the prognostic use of inflammation-based composite indices (CAR, NLR, PLR, SII, etc.), results may vary depending on the population, measurement timing, and endpoint definitions [18,19]. While some studies have reported that preoperative inflammatory markers are important in predicting mortality, others have emphasized that dynamic changes after surgery are more determinant [20]. The contribution of our study to this line of literature is the evaluation of CAR both preoperatively and postoperatively, and the comparative demonstration of the prognostic strength of these two time points. In particular, the stronger association of postoperative CAR with mortality supports that mortality after CABG represents a “dynamic biological process” and that a single preoperative measurement may not always provide sufficient risk discrimination. Within this framework, CRP reflecting the surgical stress response and inflammatory burden, and albumin reflecting the catabolic process and reserve loss, suggest that CAR better represents the combined risk profile that cannot be captured by single parameters [9,10,11,12,13,14,15,16,17].

Elevated CAR in the preoperative period suggests that the patient enters surgery on a biological background characterized by high inflammatory burden and weakened reserve [12]. This is particularly important clinically, because in some patients, despite low classical risk scores such as EuroSCORE II or STS, a predisposition to perioperative morbidity due to subclinical inflammation or nutritional deficiency may be overlooked [4]. Therefore, preoperative CAR may be considered a complementary biomarker capable of predicting complications that may develop in the early period even in the group of patients considered “low risk.” Moreover, elevated preoperative CAR is not only a prognostic signal but also represents a clinical window amenable to intervention; more detailed evaluation of the patient’s inflammatory status in the preoperative period, exclusion of possible occult infection foci, investigation and optimization—when possible—of accompanying conditions that may increase chronic inflammation (such as uncontrolled diabetes, advanced periodontitis, chronic lung infections, urinary tract infection), may contribute to more controlled management of the additional inflammatory burden after surgery. In addition, targeting reserve deficiency reflected through albumin levels, initiating nutritional support early in the preoperative process, and planning strategies to reduce catabolic response in the perioperative period may be practically guided by preoperative CAR. This approach suggests that CAR may be used not only as an index that “measures risk,” but also as a clinical tool that indicates perioperatively optimizable domains.

However, postoperative CAR incorporates not only the preoperative background but also surgical stress, CPB-related inflammation, hemodilution, impaired tissue perfusion, early infection/fever response, and the catabolic effects of the intensive care course [5,7,8,9]. Therefore, elevated postoperative CAR should be interpreted with caution. In the early postoperative period, increased CRP may primarily reflect surgical trauma, cardiopulmonary bypass-related sterile inflammation, and the acute-phase response, whereas decreased albumin may be influenced by hemodilution, capillary leakage, fluid balance, and postoperative catabolism. For this reason, a high postoperative CAR may not necessarily represent a direct causal predictor of mortality, but rather a composite signal of ongoing clinical deterioration, systemic inflammatory burden, and reduced physiological reserve. Thus, postoperative CAR should be regarded as an adjunctive risk marker that reflects the severity of the patient’s postoperative condition rather than a standalone determinant of outcome. Therefore, the higher discriminative performance of postoperative CAR is biologically plausible, but it should be interpreted as reflecting the combined effect of postoperative inflammation, hemodilution, catabolic response, and clinical severity rather than as evidence of a direct independent causal effect [21]. The fact that our results, in agreement with the literature, demonstrate that postoperative markers predict mortality better underscores the importance of the “postoperative biomarker monitoring” approach. Since events determining mortality in cardiac surgery practice often develop rapidly in the early postoperative period, biomarkers representing this period are expected to carry greater weight in clinical decision-making processes.

This study has several strengths. First, CAR is an index derived from two parameters commonly measured in clinical practice and does not require additional cost, and our results directly support clinical applicability. Second, by evaluating both preoperative and postoperative CAR, an important gap regarding prognostic timing has been addressed. Third, the study focused on an endpoint of high clinical importance such as mortality, and the analyses were supported by ROC performance and multivariable statistical models.

In conclusion, the CRP/albumin ratio is a practical and accessible biomarker associated with early mortality after CABG, integrating inflammation and nutritional/reserve status within the same index. In particular, the ability of postoperative CAR levels to predict mortality risk with higher accuracy may assist in the identification of patients at higher risk in the early intensive care period and allow personalization of follow-up/intervention strategies. The clinical use of CAR should be considered as part of a proactive approach aimed at reducing mortality after CABG, and it should be validated in different populations through multicenter and prospective studies and integrated into clinical decision algorithms.

### Limitations of the Study

This study has several limitations. First, the study was single-center and had a retrospective design. This makes it difficult to completely eliminate the possibility of selection bias and record-based measurement errors. However, this risk was attempted to be reduced by including consecutive patients and applying data cleaning/eligibility checks. The retrospective design may also result in an inability to record all clinical variables in a standardized manner with the same level of detail; this may lead to certain confounding factors not being fully represented in the analyses and may affect the magnitude of associations. Nevertheless, since the main outcome measures (mortality, CRP and albumin measurements) are objectively recorded parameters, a systematic bias of a magnitude that would change the direction of the main finding is not expected.

Second, as CRP and albumin measurements were obtained within the routine clinical workflow, it is possible that the timing of preoperative and postoperative measurements was not fully standardized across all patients. This heterogeneity may increase inter-individual variability in CAR values and may tend to weaken the statistical association. In addition, since postoperative albumin levels may be affected by factors such as hemodilution, fluid balance, and catabolic response, interpretation of CAR in the postoperative period should be made by considering these dynamics. However, this is an inevitable part of cardiac surgery practice and may also be considered a strength of the study in terms of testing the clinical applicability of CAR under “real-life conditions.”

Third, the limited number of mortality events may have restricted the number of covariates that could be simultaneously evaluated in multivariable models and may have increased the possibility of residual confounding and model overfitting despite efforts to reduce instability through restricted model construction and internal validation procedures. In addition, established surgical risk models such as EuroSCORE II and STS were not available for direct comparison in the present dataset. Therefore, the incremental prognostic contribution of CAR beyond validated perioperative risk scoring systems could not be formally evaluated. Furthermore, although SYNTAX score was included as an anatomical complexity parameter, it should not be interpreted as a surrogate for comprehensive surgical risk models. For this reason, the adjusted models should be interpreted primarily as exploratory risk-adjustment frameworks rather than definitive causal models, and external validation in larger multicenter cohorts remains necessary. In addition, the use of an event-enriched sampling strategy represents an important methodological consideration. Although this approach increases statistical power in analyses involving relatively rare outcomes such as mortality, it alters the natural event prevalence within the study cohort. This may lead to overestimation of discrimination performance and may influence the stability and generalizability of derived cut-off values. In particular, ROC-based measures such as AUC are less sensitive to prevalence; however, threshold-dependent metrics, including sensitivity, specificity, PPV, and NPV, as well as optimal cut-off determination using the Youden index, may be affected by the altered event distribution. Therefore, the identified CAR cut-off values should be interpreted with caution and should not be directly extrapolated to populations with different baseline risk profiles. Therefore, the results should be interpreted within a framework supporting a prognostic association rather than establishing a causal relationship. Accordingly, the adjusted models should be interpreted primarily as exploratory risk-adjustment frameworks rather than definitive causal models, and external validation in larger multicenter cohorts remains necessary. Accordingly, the overall findings of the present study should be considered exploratory and hypothesis-generating rather than definitive evidence supporting incorporation of CAR into routine CABG risk stratification algorithms. The cut-off values determined by ROC analysis may also be specific to the study population; since the performance of these thresholds may vary in different patient groups, external validation studies are required. In addition, the derivation of optimal cut-off values from the same dataset used for model development introduces a potential risk of optimism bias, which may lead to overestimation of diagnostic performance. Although internal validation was partially addressed using bootstrap methods, more robust validation approaches and external validation in independent cohorts are required to confirm the reproducibility and generalizability of these thresholds.

Finally, the lack of assessment of CAR trends through serial measurements is a limitation in terms of dynamic risk stratification. Nevertheless, our study demonstrates the potential of CAR, an easily accessible composite biomarker in the preoperative and early postoperative periods, for predicting mortality and provides an important signal that may form the basis for a clinically applicable risk classification approach.

## 5. Conclusions

In conclusion, CAR demonstrated moderate discriminative ability for identifying patients at increased risk of early mortality after CABG, particularly in the postoperative period. However, because its association did not remain independent after multivariable adjustment, CAR should be interpreted primarily as a complementary and exploratory inflammation-based biomarker reflecting postoperative physiological stress rather than as a stand-alone prognostic determinant. These findings support the concept that inflammation-related composite biomarkers may contribute supportive biological information alongside conventional perioperative assessment, although their incremental value beyond validated surgical risk models requires further prospective and multicenter investigation.

## Figures and Tables

**Table 1:** Baseline characteristics of the study population according to mortality status (Ex vs. Non-Ex) (*n* = 273).

Variables	Non-Ex (*n* = 232)	Ex (*n* = 41)	*p*-Value
Age, years	62.0 (58.0–67.0)	62.0 (56.8–70.0)	0.491
Sex, male, n (%)	168 (72.4)	23 (56.1)	0.055
EF, %	55.0 (50.0–57.0)	50.0 (46.0–55.0)	**0.029**
Diabetes mellitus, n (%)	112 (48.3)	21 (51.2)	0.859
Hypertension, n (%)	141 (60.8)	25 (61.0)	1.000
SYNTAX score	21.0 (17.0–24.1)	21.5 (17.0–25.4)	0.896
HbA1c, %	6.3 (5.7–7.1)	6.5 (5.8–7.8)	0.245
LDL, mg/dL	103.3 (73.0–129.3)	116.6 (83.9–145.0)	**0.044**
HDL, mg/dL	43.2 (35.0–52.0)	40.1 (33.0–53.2)	0.839
Total cholesterol, mg/dL	180.0 (137.8–212.3)	184.0 (132.0–250.0)	0.679
CPB Time	109.2 ± 26.4	162.4 ± 47.3	**<0.001**
Cross Clamp Time	54.9 ± 14.7	121.5 ± 24.6	**<0.001**

Statistical tests applied include *p* Value: Continuous variables are presented either as median (IQR) or mean ± standard deviation according to distribution characteristics. The Mann–Whitney U test was used for non-normally distributed continuous variables, whereas the independent samples Student’s *t*-test was used for normally distributed continuous variables. Categorical variables (reported as n [%]) were compared using the Chi-square test or Fisher’s exact test where appropriate. In the table, statistically significant values are marked in bold. The *p*-value indicates the level of statistical significance, with values less than 0.05 considered statistically significant. Ex = Mortality group, Non-Ex = Non-mortality group, EF = Ejection Fraction, SYNTAX = Synergy Between PCI With TAXUS and Cardiac Surgery score, HbA1c = Glycated hemoglobin, LDL = Low-density lipoprotein, HDL = High-density lipoprotein, CPB = Cardiopulmonary bypass.

**Table 2:** Preoperative laboratory and inflammatory markers according to mortality (*n* = 273).

Variables	Non-Ex (*n* = 232)	Ex (*n* = 41)	*p*-Value
**Preoperative inflammatory and nutritional markers**			
CRP, mg/L	31.7 (13.0–68.0)	62.0 (21.0–123.8)	**0.007**
Albumin, g/L	34.0 (31.3–37.0)	32.0 (29.0–35.0)	**0.009**
CAR (CRP/albumin)	0.91 (0.39–2.19)	1.85 (0.67–4.33)	**0.003**
**Preoperative renal and electrolyte parameters**			
Urea, mg/dL	35.0 (27.0–46.8)	45.0 (33.5–70.0)	**0.001**
Creatinine, mg/dL	0.88 (0.76–1.04)	1.07 (0.82–1.57)	**0.005**
GFR, mL/min/1.73 m^2^	84.0 (66.0–101.0)	66.4 (42.0–91.0)	**0.009**
Sodium, mmol/L	139.0 (137.0–141.0)	138.0 (135.0–140.0)	0.149
Potassium, mmol/L	4.3 (4.1–4.6)	4.4 (4.0–4.8)	0.363
**Preoperative hematological parameters**			
Hemoglobin, g/dL	13.3 (12.3–14.3)	12.6 (10.9–13.7)	**0.016**
Hematocrit, %	40.4 (37.1–43.4)	38.7 (33.4–41.1)	**0.023**
WBC, ×10^9^/L	8.7 (7.3–10.6)	10.1 (7.8–12.6)	**0.039**
Platelets, ×10^9^/L	245.0 (201.0–293.0)	231.0 (172.5–278.5)	0.267
RDW, %	14.3 (13.6–15.2)	14.9 (14.2–16.2)	**0.006**
PDW, %	16.5 (15.7–17.3)	16.7 (15.8–17.6)	0.469
IG, %	0.50 (0.32–0.82)	0.60 (0.40–1.10)	0.098
MPV, fL	9.9 (9.2–10.6)	9.8 (9.1–10.9)	0.866
Monocytes, ×10^9^/L	0.60 (0.47–0.77)	0.71 (0.49–0.96)	0.096
Lymphocytes, ×10^9^/L	1.52 (1.15–1.96)	1.29 (0.88–1.82)	0.098

Statistical tests applied include *p* Value: Mann–Whitney U test for non-normally distributed continuous variables (reported as median [IQR]) and the Chi-square test or Fisher’s exact test for categorical variables where applicable. In the table, statistical values that are significant are marked in bold. The *p*-value indicates the level of statistical significance, where values less than 0.05 are considered significant. Ex = Mortality group, Non-Ex = Non-mortality group, CRP = C-reactive protein, CAR = CRP-to-albumin ratio, GFR = Glomerular filtration rate, WBC = White blood cell, RDW = Red cell distribution width, PDW = Platelet distribution width, IG = Immature granulocytes, MPV = Mean platelet volume.

**Table 3:** Postoperative laboratory parameters according to mortality (*n* = 273).

Variables	Non-Ex (*n* = 232)	Ex (*n* = 41)	*p*-Value
**Postoperative inflammatory and nutritional markers**			
CRP, mg/L	42.5 (21.0–83.1)	94.0 (40.0–153.0)	**<0.001**
Albumin, g/L	29.0 (27.0–32.0)	27.0 (24.8–30.0)	**0.001**
CAR (CRP/albumin)	1.44 (0.73–2.93)	3.39 (1.55–6.18)	**<0.001**
**Postoperative renal parameters**			
Urea, mg/dL	34.0 (25.0–47.0)	59.0 (39.0–97.0)	**<0.001**
Creatinine, mg/dL	0.89 (0.76–1.06)	1.63 (1.00–2.62)	**<0.001**
GFR, mL/min/1.73 m^2^	82.2 (64.0–99.0)	46.0 (24.9–74.0)	**<0.001**
**Postoperative hematological parameters**			
Hemoglobin, g/dL	10.6 (9.7–11.8)	9.8 (8.5–11.2)	**0.018**
Hematocrit, %	32.3 (29.4–36.4)	30.1 (26.1–34.2)	0.068
WBC, ×10^9^/L	11.4 (9.3–13.8)	13.0 (10.4–16.5)	**0.009**
Platelets, ×10^9^/L	186.0 (142.0–239.0)	158.0 (106.0–204.0)	**0.024**
RDW, %	14.4 (13.7–15.3)	15.4 (14.6–16.9)	**<0.001**
PDW, %	16.6 (15.8–17.5)	16.8 (15.9–17.9)	0.461
MPV, fL	10.0 (9.3–10.8)	10.2 (9.1–11.0)	0.736
IG, %	0.60 (0.40–0.98)	0.90 (0.60–1.50)	**0.002**
Monocytes, ×10^9^/L	0.76 (0.60–0.97)	0.83 (0.63–1.18)	0.115
Lymphocytes, ×10^9^/L	1.10 (0.84–1.49)	0.90 (0.63–1.24)	**0.015**

Statistical tests applied include *p* Value: Mann–Whitney U test for non-normally distributed continuous variables (reported as median [IQR]) and the Chi-square test or Fisher’s exact test for categorical variables where applicable. In the table, statistical values that are significant are marked in bold. The *p*-value indicates the level of statistical significance, where values less than 0.05 are considered significant. Ex = Mortality group, Non-Ex = Non-mortality group, CRP = C-reactive protein, CAR = CRP-to-albumin ratio, GFR = Glomerular filtration rate, WBC = White blood cell, RDW = Red cell distribution width, PDW = Platelet distribution width, MPV = Mean platelet volume, IG = Immature granulocytes.

**Table 4:** Univariate logistic regression analysis for predictors of postoperative mortality (*n* = 273).

Predictor	OR (95% CI)	*p*-Value
**Inflammatory and nutritional markers**		
Preoperative CAR (per 1.0)	2.00 (0.91–4.37)	0.081
Postoperative CAR (per 1.0)	1.123 (0.958–1.318)	0.153
Preoperative CRP, mg/L (per 10)	1.017 (0.994–1.040)	0.141
Preoperative albumin, g/L (per 1)	0.926 (0.857–1.000)	0.051
Postoperative CRP, mg/L (per 10)	1.011 (0.941–1.087)	0.759
Postoperative albumin, g/L (per 1)	0.862 (0.791–0.939)	**<0.001**
**Clinical and operative parameters**		
Ejection fraction, % (per 1)	0.949 (0.905–0.996)	**0.033**
Age, years (per 1)	0.991 (0.960–1.023)	0.587
SYNTAX score (per 1)	1.000 (0.950–1.053)	0.999
HbA1c, % (per 1)	1.128 (0.933–1.364)	0.213
**Renal parameters**		
Preoperative creatinine, mg/dL (per 1)	2.884 (1.047–7.941)	**0.040**
Preoperative GFR, mL/min/1.73 m^2^ (per 10)	0.996 (0.987–1.006)	0.442
Postoperative creatinine, mg/dL (per 1)	2.813 (1.466–5.395)	**0.002**
Preoperative urea, mg/dL (per 10)	1.196 (0.954–1.500)	0.121
Postoperative urea, mg/dL (per 10)	1.348 (1.101–1.652)	**0.004**
**Hematological parameters**		
Preoperative RDW, % (per 1)	1.023 (0.960–1.089)	0.485
Postoperative RDW, % (per 1)	1.069 (0.974–1.174)	0.162
Preoperative PDW (per 1)	0.987 (0.933–1.045)	0.653
Postoperative WBC, ×10^9^/L (per 1)	1.072 (0.987–1.165)	0.099
Postoperative platelets, ×10^9^/L (per 10)	0.928 (0.871–0.988)	**0.020**
Postoperative IG%, % (per 0.1)	1.000 (0.986–1.013)	0.956

Statistical tests applied include *p* Value: Univariate logistic regression analysis was performed to evaluate predictors of postoperative mortality. Odds ratios (ORs) are presented with 95% confidence intervals (95% CI). In the table, statistical values that are significant are marked in bold. The *p*-value indicates the level of statistical significance, where values less than 0.05 are considered significant. OR = Odds Ratio, CI = Confidence Interval, CAR = CRP-to-albumin ratio, EF = Ejection Fraction, GFR = Glomerular filtration rate, CRP = C-reactive protein, HbA1c = Glycated hemoglobin, RDW = Red cell distribution width, PDW = Platelet distribution width, WBC = White blood cell, IG = Immature granulocytes.

**Table 5:** Multivariate logistic regression analysis for independent predictors of mortality (*n* = 273).

Predictor	Adjusted OR	95% CI	*p*-Value
**Model 1: Preoperative adjusted model**			
Age	0.986	0.954–1.020	0.408
Female sex	2.809	1.343–5.874	**0.006**
EF	0.956	0.909–1.006	0.081
Diabetes mellitus	1.021	0.500–2.085	0.954
Hypertension	0.913	0.441–1.890	0.807
SYNTAX score	0.997	0.944–1.052	0.906
Preoperative creatinine	3.271	1.119–9.561	**0.030**
Preoperative CAR	1.899	0.806–4.470	0.142
**Model 2: Postoperative adjusted model**			
Age	0.984	0.951–1.018	0.346
Female sex	2.818	1.338–5.933	**0.006**
EF	0.952	0.904–1.002	0.058
Diabetes mellitus	0.894	0.427–1.871	0.766
Hypertension	0.896	0.430–1.866	0.769
SYNTAX score	1.009	0.956–1.065	0.743
Postoperative creatinine	3.118	1.560–6.231	**0.001**
Postoperative CAR	1.168	0.985–1.386	0.075

Statistical tests applied include *p* Value: Multivariate logistic regression analysis was performed to identify independent predictors of postoperative mortality. Adjusted odds ratios (Adjusted ORs) are presented with 95% confidence intervals (95% CI). In the table, statistical values that are significant are marked in bold. The *p*-value indicates the level of statistical significance, where values less than 0.05 are considered significant. OR = Odds Ratio, CI = Confidence Interval, CAR = CRP-to-albumin ratio, EF = Ejection Fraction, SYNTAX = Synergy Between PCI With TAXUS and Cardiac Surgery score.

**Table 6:** Discriminative performance of CAR for predicting mortality (*n* = 273).

**(A)** **ROC Analysis**					
**Parameter**		**AUC (95% CI)**			***p*-Value**
Preoperative CAR		0.676 (0.591–0.760)			**0.001**
Postoperative CAR		0.792 (0.725–0.859)			**<0.001**
EF alone		0.641 (0.555–0.726)			**0.006**
SYNTAX score alone		0.512 (0.420–0.604)			0.812
Pre CAR + EF (mini model)		0.724 (0.643–0.805)			**<0.001**
Post CAR + EF (mini model)		0.823 (0.761–0.884)			**<0.001**
**(B)** **Optimal cut-off values and diagnostic performance (Youden index)**					
**Parameter**	**Cut-off**	**Sensitivity (%)**	**Specificity (%)**	**PPV (%)**	**NPV (%)**
Preoperative CAR	**≥1.30**	63.4	67.2	25.5	91.3
Postoperative CAR	**≥2.60**	78.0	74.6	36.5	94.6
**(C)** **Binary risk estimation using CAR cut-off**					
**Parameter**	**OR (95% CI)**				***p*** **-value**
Pre CAR ≥ 1.30	3.12 (1.48–6.56)				**0.003**
Post CAR ≥ 2.60	6.85 (3.20–14.65)				**<0.001**

Statistical tests applied include *p* Value: ROC curve analysis was used to evaluate the discriminative ability of CAR and the derived mini-models, with performance reported as AUC with 95% confidence intervals (95% CI). Optimal cut-off values were determined using the Youden index, and diagnostic performance was reported as sensitivity, specificity, positive predictive value (PPV), and negative predictive value (NPV). Binary risk estimation for mortality according to CAR cut-off values was assessed using logistic regression and presented as OR with 95% CI. In the table, statistical values that are significant are marked in bold. The *p*-value indicates the level of statistical significance, where values less than 0.05 are considered significant. ROC = Receiver operating characteristic, AUC = Area under the curve, CI = Confidence Interval, CAR = CRP-to-albumin ratio, EF = Ejection Fraction, SYNTAX = Synergy Between PCI With TAXUS and Cardiac Surgery score, OR = Odds Ratio, PPV = Positive predictive value, NPV = Negative predictive value.

**Table 7:** Calibration performance of multivariable models for predicting in-hospital mortality.

Model	AUC (95% CI)	Calibration Slope	Calibration Intercept	Brier Score
Preoperative model (Clinical + Pre-CAR)	0.688 (0.603–0.773)	0.91	0.04	0.136
Postoperative model (Clinical + Post-CAR)	0.730 (0.651–0.809)	0.95	0.02	0.129

Calibration performance of the multivariable logistic regression models was evaluated using calibration slope, calibration intercept, and Brier score. Calibration slope values close to 1 indicate optimal agreement between predicted and observed risks, while values < 1 suggest potential overfitting. Calibration intercept values close to 0 indicate absence of systematic over- or underestimation of risk. The Brier score reflects overall model accuracy, with lower values indicating better predictive performance. AUC values represent discrimination ability and are presented alongside calibration metrics for comprehensive model evaluation.

## Data Availability

The data that support the findings of this study are available from the corresponding author upon reasonable request. The data are not publicly available due to privacy and ethical restrictions.

## Abbreviations

CAR	C-reactive protein-to-albumin ratio
CABG	Coronary artery bypass grafting
CRP	C-reactive protein
ROC	Receiver operating characteristic
AUC	Area under the curve
CI	Confidence Interval
